# Disorders of gut–brain interaction through the lens of polyvagal theory

**DOI:** 10.1111/nmo.14926

**Published:** 2024-09-30

**Authors:** Stephen W. Porges

**Affiliations:** ^1^ Indiana University Bloomington Bloomington Indiana USA; ^2^ University of North Carolina at Chapel Hill Chapel Hill North Carolina USA

**Keywords:** autonomic nervous system, disorders of gut–brain interaction, neurogastroenterology, polyvagal theory, vagal efficiency, ventral vagal complex

## Abstract

This paper introduces a metric capable of tracking a hypothetical brainstem “switching” mechanism involved in regulating the afferent influence of blood pressure on the vagal efferent control of heart rate. In theory, this metric could be applied to evaluate the “efficiency” of brainstem pathways involved in common mechanisms of autonomic function involving the vagal influences on the gut as well as the heart. Thus, by exploring the dynamic “efficiency” of the brainstem feedback circuit linking heart rate to posture, a clinically relevant index of vagal flexibility might be extracted that would provide a generalizable window into the vagal regulation of both the heart and gut. Recent research supports this contention and has documented that this metric, VE, appears to covary with disorders of the gut. Clinical application of this metric might identify individual vulnerabilities that frequently reflect symptoms assumed to have features of a dysregulated autonomic nervous system (i.e., dysautonomia). If this is confirmed by additional research, then this objective measure of neural regulation of autonomic function might provide insight into the pathogenesis of disorders of gut–brain interaction.

## Graphical abstract

This paper introduces a metric, vagal efficiency (VE), that tracks a brainstem “switching” mechanism regulating blood pressure's effect on vagal control of the heart and gut. Potentially offering insights into gut–brain interaction disorders.

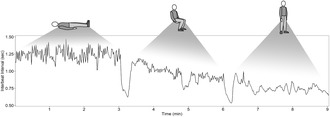

**Key points**

- Modern healthcare often focuses on symptoms without addressing underlying neural mechanisms, which can lead to fragmented care and missed root causes.
- Prioritizing symptom relief without addressing underlying neural issues can lead to misdiagnoses, inappropriate treatments, and prolonged mediciine use.
- Monitoring a novel metric of dynamic autonomic regulation, vagal efficiency, is proposed to offer valuable insights into the autonomic nervous system’s role in promoting optimal gut function and general health.

## 1 INTRODUCTION

Modern medicine has made significant progress in diagnosing and treating diseases, yet it often relies heavily on a symptom‐based model. While this approach has merits, it also possesses limitations, especially when attempting to infer underlying neural mechanisms involved in the expression of a disease. This is a common problem in gastroenterology, in which disease entities are, in general, based on symptom clusters often dependent on a mixture of subjective reports of pain or discomfort and objective observable indices of gut motility. These forms of assessment are secondary to potential causal influences of the neural pathways involved in regulating the gut. Thus, traditional clinical assessments dependent on human subjective experience and even peripheral autonomic measures may reframe and obscure potentially causal neural mechanisms. For example, expectancies and associations of visceral feelings assessed by subjective responses may be dependent on contextual experiences leading to top‐down changes in the gut due to expectancies, associative learning, classical conditioning, and memories.

A primary criticism of the symptom‐based model is its tendency to prioritize symptom relief over addressing underlying disease mechanisms, leaving the root causes unaddressed. For instance, painkillers may temporarily alleviate chronic pain but fail to tackle inflammatory conditions, neural regulation disruptions, or structural issues as potential root causes. Furthermore, the symptom‐based model contributes to a fragmented healthcare system where specialists focus on isolated symptoms, neglecting the patient's neural regulation of the entire autonomic nervous system. This lack of coordination can result in a disjointed approach to healthcare, leading to misdiagnoses, mislabeling of a general autonomic disruption involving several target organs as comorbidities, treatment interactions, delays in appropriate treatment, and suboptimal outcomes. Clinically, this approach perpetuates symptom management instead of disease resolution, leading to long‐term medication regimens without addressing the underlying ailment.

Historically, the symptom‐focused model is rooted in medical practice prior to innovations that have led to assays of bodily fluids and tissues, imaging techniques, and electrophysiology. This symptom‐focused model represents a medical legacy that is captured in nosology. Nosology is a pragmatic atheoretical perspective of pathology consisting of using symptoms to classify diseases without explaining underly etiology (cause) or pathogenesis (causal mechanism). In some areas of medicine in which mechanisms are hypothetical (e.g., Psychiatry), nosology dominates training and treatment models.

Despite its limitations, a focus on symptoms plays an important role in healthcare. Optimal public health strategies inform the public to be aware of symptoms and when observed to be sufficiently motivated to seek medical guidance and standardized evaluations. These evaluations, although starting with a symptom cluster, are frequently confirmed with biological assays through blood, saliva, tissue, imaging, or electrophysiological monitoring. When there are no reliable biomarkers, symptoms may become unexplainable to the physician and the disorder is frequently reclassified as a functional disorder or even viewed by the medical staff as “medically unexplained symptoms.” Thus, the decision to diagnose a patient as having a functional disorder that is not confirmed by etiology or pathogenesis is pragmatically dependent on the current state of both the technology applied to assess and confirm a specific purported pathway to illness or the plausible theory relating the observable symptoms to the disease.

The gap between observable symptoms and reliable assessments documenting pathogenesis is frequently observed in gastroenterology. Although gastrointestinal symptoms are highly prevalent and disrupt quality of life, approximately 40% have no organic explanation for their symptoms.^1^, ^2^, ^3^, ^4^ Currently most patients, who have symptoms that do not converge with the generally available medical assessments, are labeled as having a functional gastrointestinal disorder or more accurately disorders of gut–brain interaction (e.g., irritable bowel syndrome and functional dyspepsia). Historically, patients with a “functional” diagnosis may have been dismissed by physicians are not having a “valid” disorder and may have been referred to a psychiatrist or psychologist, implying that the patient has a psychological problem and not a valid disease. At best, the patient would leave the clinic with a sense that their discomfort was due to external signals that promote stress and anxiety independent of any neurophysiological processes that would implicate the gut.

Pragmatically, from a data collection and research paradigm perspective, it is easier to collect and assay biological samples or to view static images from X‐rays and MRIs than to be trained to generate metrics that accurately assess the dynamic neural regulation of the end organ of interest (e.g., gut). Although technological advances have enhanced the ability to monitor the physiology of gastrointestinal processes (e.g., electrogastrogram, MRI), clinical applications have been limited due to availability, training, and cost. In contrast, wearable technologies have impacted on consumer interest in heart rate and heart rate variability, but not gut function. The advance in sensors and signal processing has led to low‐cost wearables enabling continuous monitoring of cardiac function outside the constraints of the clinic. However, these technologies, not only have overlooked the gut, but also have overlooked refinement of indices of neural function. The technologies, by being focused primarily on monitoring peripheral physiological signals of the autonomic nervous system, such as heart rate, have only crudely and often inaccurately inferred neural regulation by quantifying heart rate variability. This strategy has limited the flow of information to the patient and physician regarding the dynamic “neural” feedback circuits involving brainstem regulation of the efferent pathways reflected in peripheral physiological signals.^5^, ^6^

Medical specialties are organ focused, resulting in disciplines that study the organ and not the neural regulation of the organ. In addition, when there is a more general “system” dysfunction in neural regulation, this strategy may result in the appearance of dysfunction in more than one organ (i.e., comorbidities). Frequently, the assessment of an organ, without a concrete metric of disruption of function, assumes that the disorder is not physiologically based and is solely psychological. This conclusion limits the support and treatment that the clinician can provide and places the patient at risk. Several disorders have been assumed to have psychological components, since the neural pathways are not known, and the intensity of symptoms is frequently linked to stressful situations. Fortunately, this portrayal of the treatment and understanding of functional disorders is changing with new “neural” oriented specialties such as neurogastroenterology^7^, ^8^ that have emerged in response to the paucity of information and theoretical conceptualization involving the causal role of the nervous system and especially the vagus in pathogenesis.

A primary objective for “integrating” disciplines, such as neurogastroenterology, is to objectively describe the relationship between the nervous system and visceral organs. However, for those who study the neural regulation of the gut and other visceral organs, there is a shared knowledge that brain structures and peripheral organs are interconnected through neural pathways including the vagus that send bidirectional signals between visceral organs and the brainstem. These bidirectional communication circuits provide dynamic regulatory mechanisms through which brain structures influence visceral organs and visceral organs inform and influence brain function. This premise is the basis of Polyvagal Theory^9^, ^10^ and an important assumption in neurogastroenterology. Fortunately, the emerging interest the bidirectional communication between visceral organs and the brain, especially with an interest in the role of the vagus is changing the research and clinical culture dealing with gut disorders. For example, the focus of this special issue of *Neurogastroenterology and Motility* emphasizes the shifting emphasis of the role of the brain and the bidirectional neural communication between the brain and gut as a plausible determinant of gut disorders.

## 2 EMPHASIS ON VAGAL NEURAL PATHWAYS

Applications of the electrogastrogram are based on the validity of the myoelectric activity being driven by the intrinsic neural networks within the stomach that generate spontaneous slow rhythmic electrical activity mediated by vagal pathways. Research has documented that noninvasive transcutaneous auricular vagal nerve stimulation enhances gastric slow wave activity.^11^ These methods add objective validation of vagal mechanisms being involved in the efferent pathways co‐occurring with symptoms.

The vagus is the cranial nerve that contains the primary parasympathetic pathways to and from virtually all visceral organs. Vagal pathways support the homeostatic functions of visceral organs including the heart and gut via more efficient digestion and flow of oxygenated blood through the body. In contrast, the sympathetic nervous system may be viewed as disruptive to homeostasis, while supporting metabolically costly mobilization processes that may be expressed as fight or flight behaviors and other more extensive defensive reactions to predator and pathogen that would include inflammation, fever, and gastroparesis. This simplistic dichotomy is, in general, a useful model explaining the changes in autonomic state, when an organism is in a safe environment or is transitorily confronted with challenges such as threat that would require an increase in metabolic output to fight or flee. This model has led to a concept of autonomic balance and a generalized assumption that the parasympathetic (including the vagus) portion of the autonomic nervous system supports homeostatic needs, while the sympathetic nervous system supports defensive needs including responding to signals of stress and threat.

Through the lens of the Polyvagal Theory (see below), the synergistic model of autonomic balance focusing on the efferent pathways and consisting of two antagonistic components (i.e., parasympathetic and sympathetic) is too limited. The traditional model by neglecting the importance of afferent pathways and brainstem structures involved in the dynamic regulation of end organs may lead to faulty inference regarding potential pathogenesis. For example, within the gastroenterology literature, the role of the vagus in the production of ulcers has been described as a paradox,^12^ because the vagus has been implemented in both optimizing digestion and in the production of ulcers. Polyvagal Theory provides a lens to interpret the vagal paradox described by Burge as well as several functional disorders of the gut as being due to predictable changes in neural regulation that would disrupt function prior to end organ damage. There is also the possibility a vagal defensive reaction, as observed by Burge, may also confound the interpretation of slow wave electrogastrogram activity as vagal influences on the gut may not be linear.

## 3 POLYVAGAL THEORY

Polyvagal Theory emerged as a solution to another vagal paradox,^9^, ^10^ one in which vagal activity to the heart was conceptualized as calming and supporting health, while the other was potentially lethal. In heart rate, the beneficial influence was observed as the respiratory component of heart rate variability and the detrimental influence was observed as bradycardia.

Identifying the vagal mechanisms underlying the paradox evolved into the “Polyvagal Theory.” In developing the theory, the anatomy, development, evolutionary history, and function of the two vagal systems were identified as follows: one system was potentially lethal, while the other system was protective. The two vagal pathways originated in different areas of the brainstem. Through the study of comparative anatomy, it can be inferred that the two vagal circuits evolved sequentially.^13^ This sequence was further observed during mammalian embryology and early postnatal development.^14^

Hypotheses driven by Polyvagal Theory are related to the documentation that the reactivity of the mammalian autonomic nervous system is hierarchically organized based on phylogeny that is mirrored in embryological development. This fact became a core principle upon which Polyvagal Theory informed hypotheses could be tested. This emphasis on hierarchy is focused on autonomic reactivity and does not preclude chronic optimal homeostatic states that are dependent on a functional balance between more systemic parasympathetic and sympathetic influences throughout the autonomic nervous system.

Early during vertebrate phylogeny (e.g., jawless fish), efferent pathways emerged from a dorsal area of the brainstem known as the dorsal motor nucleus of the vagus (DMX). Following the emergence of the dorsal vagus, a spinal sympathetic nervous system (SNS) evolved in bony fish. With its broad scope and target specificity and integration into the skeletal‐motor system, the SNS provided a rapid and coordinated fight/flight system for mobilizing the body in response to threats. Phylogenetically, the newest component of the autonomic system is the ventral vagal complex (VVC), which emerged during the transition from primitive extinct reptiles to mammals. Like the SNS before it, this system built on the integration of afferent information arising from the body and descending signals from higher brain structures to respond to internal and external needs. Its efferent arm emerged as cell bodies of vagal efferent source nuclei of the DMX located in the dorsal area of the brainstem migrated ventrally, forming a second and distinct cardioinhibitory nucleus known as the nucleus ambiguus (NA) and became integrated with circuits that regulate the bronchi and muscles of the face and head via special visceral efferent pathways. Unlike earlier systems, which promoted defense‐related survival responses, this new autonomic face‐heart connection was formed in concert with the mammalian dependence on social‐affiliative behaviors. It provided a substrate for ingestion (e.g., nursing) and co‐regulation of biobehavioral states through communication of accessibility via vocalizations and facial expressions. Since the components of the autonomic nervous system function in a phylogenetically organized hierarchy, the VVC has the capacity to dampen older survival‐based response systems.

Depending on the state of the ventral vagus, autonomic regulation may either function hierarchically or synergistically. When the ventral vagal circuit is active, it literally constrains the sympathetic nervous system to keep it from fully being expressed, as it is during fight‐flight reactions to threats. During calm and social states characterized by an active ventral vagal state, there is likely to be a synergistic coordination between dorsal vagal and sympathetic pathways (i.e., autonomic balance) optimizing the regulation of the subdiaphragmatic process associated with digestion.

## 4 VAGAL BRAKE AND VAGAL EFFICIENCY

In humans, the ventral vagal efferent pathways to the heart function as a brake. The intrinsic rate of the heart in the healthy human, even without sympathetic excitation, is significantly faster than the resting heart rate. Thus, under most conditions, the vagus, primarily via myelinated vagal fibers originating in the nucleus ambiguus, actively inhibit heart rate. However, when there is a need to engage actively with select elements in the environment, neurons originating at higher levels inhibit the brainstem regulation of homeostatic needs, and cardiac output is rapidly increased to match metabolic demands.

Under these situations, there is a transitory withdrawal of the vagal tone to the heart to increase heart rate, which defines the removal of the vagal brake.^15^ The vagal brake represents the actions of engaging and disengaging the ventral vagal influence on the heart's pacemaker. In addition, the release of the vagal brake on the heart also enables tonic underlying sympathetic excitation to exert more influence on the autonomic nervous system and inhibit gut motility.

Polyvagal Theory does not interpret homeostasis as being locked to a set point but is more consistent with the construct of allostasis,^16^, ^17^, ^18^ which also emphasizes the dynamic adjustment of autonomic function to match the metabolic demands of behavior. However, Polyvagal Theory focuses on the specific role of the ventral vagal brake as an important neural pathway in serving this purpose.

Vagal efficiency (VE), as a metric, evaluates the dynamic functional impact of efferent vagal fibers on heart rate. In mammals, chronotropic vagal influences are primarily conveyed via efferent fibers originating in nucleus ambiguus, the source nucleus of the ventral vagus, that terminate on the sinoatrial node. This relationship has led scientists to assume that heart rate, in the absence of sympathetic excitation, would be tightly coupled with dynamic changes in vagal tone. However, the ventral vagal pathway to the heart is more complex and the dynamic vagal influence on the pacemaker is influenced by brainstem mechanisms. For example, brainstem mechanisms producing a respiratory rhythm functionally gate the efferent vagal influence on the heart and produce a dynamically changing transitory respiratory pattern in heart rate known as respiratory sinus arrhythmia,^19^ RSA is functionally the modulation of the myelinated ventral vagal fibers by a brainstem cardio‐pulmonary oscillator.^20^

The link between RSA and cardiac vagal tone has a long history going back to Hering (1910),^21^ who identified a respiratory rhythm in the vagal cardioinhibitory fibers traveling from the brainstem down to the heart. Hering's observation and the more contemporary neurophysiological research^13^, ^20^ provided scientists with a neurophysiological basis to develop noninvasive methods for measuring vagal activity using the RSA component of heart rate variability and to test hypotheses generated by the Polyvagal Theory.

**FIGURE 1:**
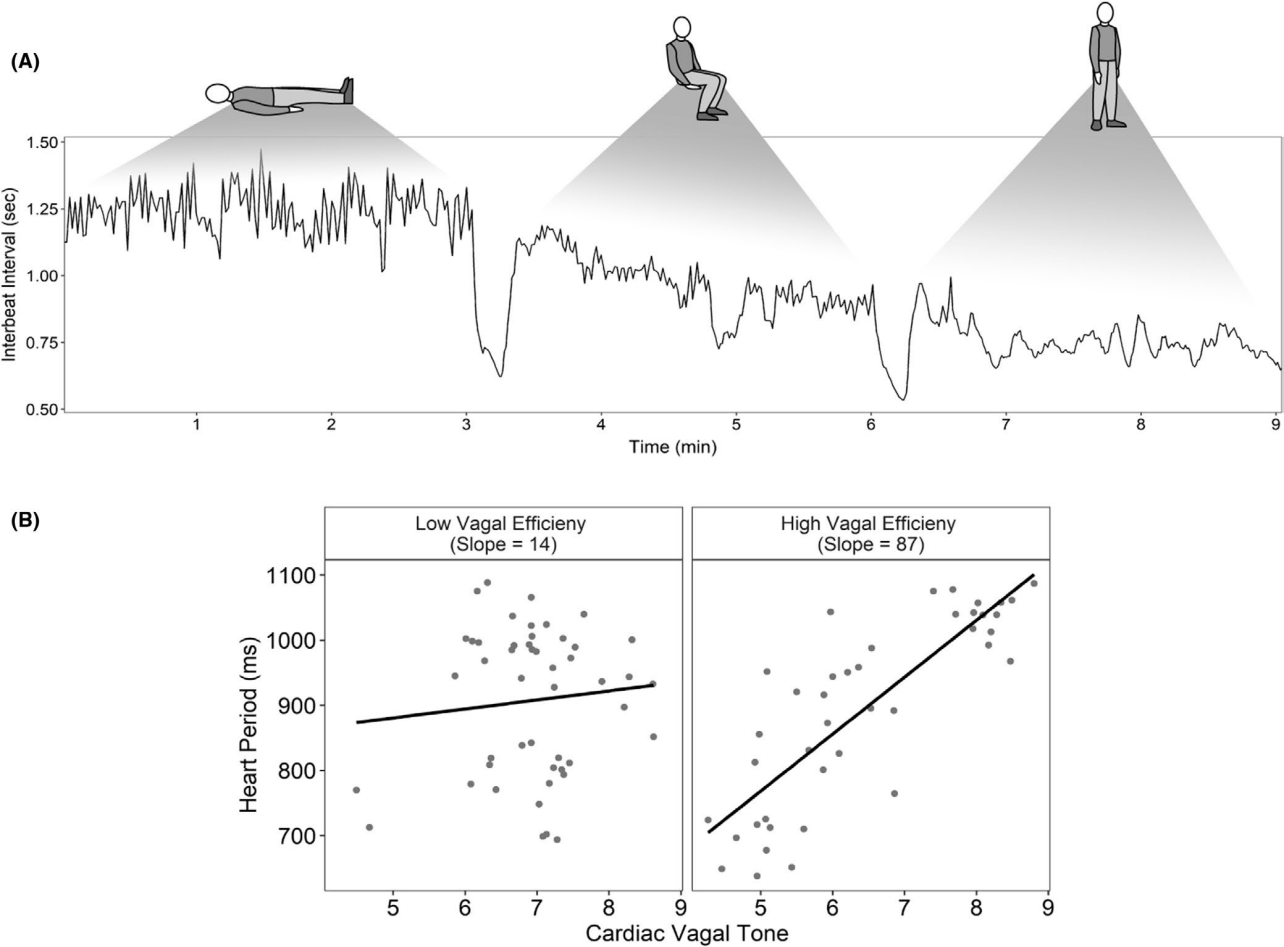
Example of vagal efficiency defined by the slope of synchronous measures of respiratory sinus arrhythmia (RSA) and heart period measured over posture changes (A). Though both adolescents have a similar range of vagal tone values (measured by RSA, panel B), the adolescent on the left has low vagal efficiency (slope = 14), suggesting that a vagal brake has a weak effect on cardiac output. The adolescent on the right has high vagal efficiency (slope = 87), suggesting that changes in cardiac vagal tone have a stronger effect on dynamic changes in heart period. Reprinted from Kolacz et al.^25^

In the early 1980s, my laboratory introduced a time–frequency methodology to quantify RSA.^22^, ^23^ The index of cardiac vagal tone generated by this methodology had several advantages including: (1) it provided a more accurate estimate of cardiac vagal tone than other methods,^24^ and (2) it could be quantified during short time periods (e.g., 10–15 s). The latter point enabled an opportunity to ask new questions and test new hypotheses that required the evaluation of dynamic changes in vagal efferent activity. For example, the methodology enabled quantification of a new metric, vagal efficiency (VE), that could index the dynamic relationship between RSA and heart rate during a physiological challenge (e.g., posture shift). As illustrated in Figure 1, VE can be visually represented as a regression line and objectively quantified as the slope depicting the ms change in heart period that would occur with a change of one log unit of RSA amplitude (i.e., the units of quantification defined in the Porges‐Bohrer methodology).^24^, ^25^

The initial study documented that sleep state in newborn infants could be reliably detected by quantifying VE.^26^ This finding has been replicated with high‐risk preterm infants and expanded to index clinical course.^27^ Additional studies indicated that in response to alcohol, VE decreased in humans.^28^ As we became more familiar with the metric, systematic challenges were used. In a clinical study using a posture challenge protocol to functionally stimulate baroreceptors, it was possible to detect a subset of patients in a pediatric gastroenterology clinic with joint hypermobility syndrome.^25^ In another study, an exercise bike challenge was used in a study with college students and VE distinguished participants with and without a maltreatment history.^29^ Most relevant to this special issue, is the documentation that children with cyclic vomiting syndrome have atypically low VE.^30^ On a clinical level, it appears that VE may provide an objective metric related to the clinical features associated with a diagnosis of dysautonomia.

Previous research documented the robust sensitivity of RSA using the Porges‐Bohrer method, to cholinergic blockade.^24^, ^31^, ^32^ In contrast, VE was insensitive to partial cholinergic blockade, when calculated from the data reported in the study.^24^ Thus, it appears that VE, unlike the robust sensitivity of RSA to peripheral vagal tone originating in the nucleus ambiguus, maybe assessing a brainstem regulation mechanism. Convergent with that hypothesis, our research has documented that VE provides clinically relevant information not observed in measures of RSA or heart rate. For example, we documented that VE, but not RSA or heart rate, mediated the clinical effectiveness of auricular vagal nerve stimulation and discriminated the hypermobility subtype of Ehlers‐Danlos Syndrome and cyclic vomiting syndrome from healthy controls.

Based on our preliminary research, VE appears to be lower in individuals with DGBI. Future research will determine whether the VE metric will provide a reliable objective measure of the vagal contribution to DBGI. Although VE seems to be a useful metric for detecting atypical vagal regulation, future research will need to be conducted to document the scope of specific DGBI diagnoses that covary with depressed VE. Moreover, future research will need to document the stability and the impact of treatment for DGBI on this metric.

In summary, VE may be a powerful, low cost, easily quantifiable, and scalable measure that would potentially provide rapid throughput screening that would identify a ventral vagal parameter of atypical autonomic regulation. The VE metric might contribute to refined diagnoses of dysautonomia and several functional disorders including DGBI. Hypothetically, this metric could be applied to evaluate the “efficiency” of brainstem pathways involved in common mechanisms of autonomic function involving the vagal influences on the gut as well as the heart. Thus, by exploring the dynamic “efficiency” of the brainstem feedback circuit linking heart rate to posture, a clinically relevant index of vagal flexibility might be extracted that would provide a generalizable window into the vagal regulation of both the heart and gut. Such a metric might identify individual vulnerabilities that frequently reflect symptoms assumed to have features of a dysregulated autonomic nervous system (i.e., dysautonomia). If this is confirmed by additional research, then this objective measure of neural regulation of autonomic function might provide insight into the pathogenesis of DGBI.

## AUTHOR CONTRIBUTIONS

Stephen W. Porges is the sole author of this manuscript and is solely responsible for the conceptualization, writing, methodology, and representation of all intellectual information described in the manuscript.

## CONFLICT OF INTEREST STATEMENT

The author declares no conflicts of interest.

## Data Availability

Data sharing not applicable to this article as no datasets were generated or analysed during the current study.

## References

[1] Chang L . Review article: epidemiology and quality of life in functional gastrointestinal disorders. Aliment Pharmacol Ther. 2004;20(s7):31‐39. doi:10.1111/j.1365-2036.2004.02183.x 15521853

[2] Mohaghegh Shalmani H , Soori H , Khoshkrood Mansoori B , et al. Direct and indirect medical costs of functional constipation: a population‐based study. Int J Color Dis. 2011;26(4):515‐522. doi:10.1007/s00384-010-1077-4 20957375

[3] Talley NJ . Functional gastrointestinal disorders as a public health problem. Neurogastroenterol Motil. 2008;20(s1):121‐129. doi:10.1111/j.1365-2982.2008.01097.x 18402649

[4] Talley NJ , Boyce P , Jones M . Predictors of health care seeking for irritable bowel syndrome: a population based study. Gut. 1997;41(3):394‐398. doi:10.1136/gut.41.3.394 9378398 PMC1891476

[5] Porges SW . Heart rate variability: a personal journey. Appl Psychophysiol Biofeedback. 2022;47(4):259‐271. doi:10.1007/s10484-022-09559-x 36136145 PMC9718870

[6] Porges SW . Cardiac vagal tone: a neurophysiological mechanism that evolved in mammals to dampen threat reactions and promote sociality. World Psychiatry. 2021;20(2):296‐298. doi:10.1002/wps.20871 34002521 PMC8129829

[7] Mayer EA . Gut feelings: the emerging biology of gut–brain communication. Nat Rev Neurosci. 2011;12(8):453‐466. 21750565 10.1038/nrn3071 PMC3845678

[8] Mayer EA , Nance K , Chen S . The gut–brain axis. Annu Rev Med. 2022;73(1):439‐453. 34669431 10.1146/annurev-med-042320-014032

[9] Porges SW . Orienting in a defensive world: mammalian modifications of our evolutionary heritage. A polyvagal theory. Psychophysiology. 1995;32:301‐318. doi:10.1111/j.1469-8986.1995.tb01213.x 7652107

[10] Porges SW . The vagal paradox: a polyvagal solution. *Compr* . Psychoneuroendocrinology. 2023;16(100200):1‐15. doi:10.1016/j.cpnec.2023.100200 PMC10724739 38108034

[11] Zhu Y , Xu F , Sun C . Noninvasive transcutaneous auricular vagal nerve stimulation improves gastric slow waves impaired by cold stress in healthy subjects. Neuromodulation Technol Neural Interface. 2023;26(8):1851‐1857. doi:10.1016/j.neurom.2022.03.010 35597733

[12] Burge H . A vagal paradox. The BMJ. 1970;4(5730):302‐303. doi:10.1136/bmj.4.5730.302-a 5475853 PMC1819832

[13] Gourine AV , Machhada A , Trapp S , Spyer KM . Cardiac vagal preganglionic neurones: An update. Auton Neurosci. 2016;199:24‐28. doi:10.1016/j.autneu.2016.06.003 27396874

[14] Porges SW , Furman SA . The early development of the autonomic nervous system provides a neural platform for social behaviour: a polyvagal perspective. Infant Child Dev. 2011;20(1):106‐118. doi:10.1002/icd.688 21516219 PMC3079208

[15] Porges SW , Doussard‐Roosevelt JA , Portales AL , Greenspan SI . Infant regulation of the vagal “brake” predicts child behavior problems: a psychobiological model of social behavior. Dev Psychobiol. 1996;29:697‐712. doi:10.1002/(SICI)1098-2302(199612)29:8 8958482

[16] McEwen BS , Wingfield JC . The concept of allostasis in biology and biomedicine. Horm Behav. 2003;43(1):2‐15. 12614627 10.1016/s0018-506x(02)00024-7

[17] Sterling P . Homeostasis vs allostasis: implications for brain function and mental disorders. JAMA Psychiatry. 2014;71(10):1192‐1193. 25103620 10.1001/jamapsychiatry.2014.1043

[18] Sterling P , Eyer J . Allostasis: a new paradigm to explain arousal pathology. In: Fisher S , Reason J , eds. Handbook of Life Stress, Cognition and Health. Wiley; 1988:629‐649.

[19] Eckberg DL . The human respiratory gate. J Physiol. 2003;548(2):339‐352. doi:10.1113/jphysiol.2002.037192 12626671 PMC2342859

[20] Richter DW , Spyer KM . Cardiorespiratory control. Central Regulation of Autonomic Function. Oxford University Press; 1990.

[21] Hering HE . A functional test of heart vagi in man. Menschen Munchen Med Wochenschr. 1910;57:1931‐1933.

[22] Porges SW , Bohrer RE . The analysis of periodic processes in psychophysiological research. In: Cacioppo JT , Tassinary LG , eds. Principles of Psychophysiology: Physical, Social, and Inferential Elements. Cambridge University Press; 1990:708‐753.

[23] Porges SW . Method and apparatus for evaluating rhythmic oscillations in aperiodic physiological response systems. US4510944A. 1985;4:510‐944.

[24] Lewis GF , Furman SA , McCool MF , Porges SW . Statistical strategies to quantify respiratory sinus arrhythmia: are commonly used metrics equivalent? Biol Psychol. 2012;89(2):349‐364. doi:10.1016/j.biopsycho.2011.11.009 22138367 PMC3269511

[25] Kolacz J , Kovacic K , Lewis GF , et al. Cardiac autonomic regulation and joint hypermobility in adolescents with functional abdominal pain disorders. Neurogastroenterol Motil. 2021;33:e14165. 33991431 10.1111/nmo.14165

[26] Porges SW , Doussard‐Roosevelt JA , Stifter CA , McClenny BD , Riniolo TC . Sleep state and vagal regulation of heart period patterns in the human newborn: an extension of the polyvagal theory. Psychophysiology. 1999;36(1):14‐21. 10098376 10.1017/s004857729997035x

[27] Porges SW , Davila MI , Lewis GF , et al. Autonomic regulation of preterm infants is enhanced by family nurture intervention. Dev Psychobiol. 2019;61(6):942‐952. 30868570 10.1002/dev.21841

[28] Reed SF , Gb O , David R , Porges SW . A neural explanation of fetal heart rate patterns: a test of the polyvagal theory. Dev Psychobiol. 1999;35(2):108‐118. doi:10.1002/(sici)1098-2302(199909)35 10461125

[29] Dale LP , Kolacz J , Mazmanyan J , et al. Childhood maltreatment influences autonomic regulation and mental health in college students. Front Psych. 2022;13:841749. 10.3389/fpsyt.2022.841749 PMC9201111 35722547

[30] Kolacz J , Kovacic K , Dang L , Li BU , Lewis GF , Porges SW . Cardiac vagal regulation is impeded in children with cyclic vomiting syndrome. Am J Gastroenterol. 2023;10‐14309:1268‐1275. 10.14309/ajg.0000000000002207 36716443

[31] Porges SW . Respiratory sinus arrhythmia: physiological basis, quantitative methods, and clinical implications. In: Grossman P , Janssen KHL , Vaitl D , eds. Cardiorespiratory and Cardiosomatic Psychophysiology. Springer; 1986:101‐115.

[32] Dellinger JA , Taylor HL , Porges SW . Atropine sulfate effects on aviator performance and on respiratory‐heart period interactions. Aviat Space Environ Med. 1987;58(4):333‐338. 3579820

